# Antibacterial Effect and Physical-Mechanical Properties of Temporary Restorative Material Containing Antibacterial Agents

**DOI:** 10.1155/2015/697197

**Published:** 2015-12-29

**Authors:** Amanda Mahammad Mushashe, Carla Castiglia Gonzaga, Paulo Henrique Tomazinho, Leonardo Fernandes da Cunha, Denise Piotto Leonardi, Janes Francio Pissaia, Gisele Maria Correr

**Affiliations:** ^1^Graduate Program in Dentistry, School of Dentistry, Positivo University, Rua Professor Pedro Viriato Parigot de Souza, 5300 Curitiba, PR, Brazil; ^2^Positivo University, Rua Professor Pedro Viriato Parigot de Souza, 5300 Curitiba, PR, Brazil

## Abstract

*Introduction*. For the maintenance of the aseptic chain created during the treatment the coronal sealing becomes paramount.* Aim*. Evaluating the antibacterial effect and the physical-mechanical properties of a temporary restorative material containing different antibacterial agents.* Material and Methods*. Two antibacterial agents (triclosan and chloramine T) were manually added to a temporary restorative material used as base (Coltosol). The antibacterial action of the material was analyzed using the agar diffusion method, in pure cultures of* Escherichia coli* (ATCC BAA-2336) and* Staphylococcus aureus* (ATCC 11632) and mixed culture of saliva collection. The microleakage rate was analyzed using bovine teeth, previously restored with the materials, and submitted to thermocycling, in a solution of 0.5% methylene blue, for a period of 24 hours. The physical and mechanical properties of the materials analyzed were setting time, water sorption, solubility, and compression strength.* Results*. No marginal leakage was observed for all groups. There was no statistical significant difference in antimicrobial activity, setting time, water sorption, solubility, and compression strength among the materials.* Conclusion*. The addition of antibacterial agents on a temporary restorative material did not optimize the antibacterial ability of the material and also did not change its physical-mechanical properties.

## 1. Introduction

The success of the endodontic therapy is closely related to the elimination of the microorganisms in the root canal system, through a correct biomechanical preparation [1–3]. For the maintenance of the aseptic chain created during the treatment the coronal sealing becomes paramount, avoiding marginal percolation of oral fluids and microorganisms into the system [4, 5].

Several studies indicate a direct relationship between the quality of coronal sealing and the success of endodontic treatment [4, 6–9]. The coronal leakage by restorative material (temporary or permanent) may be responsible for contamination of the canal and the appearance of periapical complications during the transoperative period [4, 5, 10, 11].

A good cervical plug allows no leakage of intracanal medication to the oral environment, ensuring the integrity of its action as intracanal dressing [12]. Furthermore, one of the main functions of temporary cements is the protection of dentin tubules exposed during removal of the smear layer, which are susceptible to the infiltration of bacteria and chemicals [13]. Thus, the temporary restorative material must have properties that prevent leakage, allow adhesion to substrates, and show good dimensional stability, mechanical strength, and antimicrobial activity [12].

The addition of antimicrobial agents to the composition of temporary cements allows conducting sanitization maintenance even if there is small bacterial infiltration via coronary access. Furthermore, an antibacterial coronal sealer could act in congruence with the canal dressings, preventing the development of remaining microorganisms, present in inaccessible locations [1, 9].

Because of its proven effectiveness, widely discussed in the literature, chlorhexidine is considered the “gold standard” and is often chosen as a positive control to verify the competence of other antimicrobial solutions [14]. An alternative agent, in an attempt to avoid the side effects of chlorhexidine, such as reversible loss of taste and staining of teeth and mucosal areas among others, is triclosan. It consists of a phenolic derivate, nonionic, broad-spectrum, antiseptic agent, which can act as both bactericidal and bacteriostatic. In the first case, it interferes with the bacterial metabolism, avoiding uptake of essential amino acids. In the second case, it acts in cell membrane permeability, allowing leaking of cytoplasm contents. Many products have been coupled in triclosan formulation in order to improve its effects, such as Gantrez and zinc citrate [14, 15].

An antiseptic substance, little used in dentistry, is chloramine T. It is an anionic, low surface tension agent, which allows interaction with other pharmaceutical formulas. Its mechanism of action is through an oxidation reaction and protein hydrolysis by changing the bacterial integrity. Its disadvantage compared to other agents refers to its low substantivity, since, in the presence of organic matter and microorganisms, this substance loses half of its effectiveness in the course of 24 hours [6, 9, 11, 16].

Although the antimicrobial capacity is of paramount importance, it is proven in the literature that the temporary restorative materials currently available in the market do not have such a property [9, 16, 17]. Thus, the objective of this study was to evaluate the antimicrobial effect of a temporary restorative material containing different agents (triclosan or chloramine T) in its composition and the possible interference of these agents on the physical and mechanical properties of the material. The hypothesis of this study is that the addition of these agents to the temporary restorative material will allow an antibacterial effect without interfering with the material's physical and mechanical properties.

## 2. Material and Methods

### 2.1. Antibacterial Activity Evaluation

The incorporation of antimicrobial agents was performed following the minimum inhibitory concentration (MIC) of each substance, which was determined by the microdilution method. The selected microorganisms were the same as used in the antibacterial activity analysis.

The antimicrobial agents (0.1% triclosan or 0.6% chloramine T) were manually added to a temporary restorative material (Coltosol, Coltène/Whaledent, Altstätten, Canton of St. Gallen, Switzerland) in a powder form. 0.02 g of triclosan or 0.12 g of chloramines T was added to each 20 g of Coltosol. Thus, the groups were described as follows: GI: Coltosol without inclusion of agents (negative control); GII: Coltosol + 0.1% triclosan; GIII: Coltosol + 0.6% of chloramine T.

The antibacterial activity of the temporary restorative material was evaluated in pure cultures of* Escherichia coli* (ATCC (American Type Culture Collection) BAA-2336) and* Staphylococcus aureus* (ATCC 11632) on a McFarland tube #1 scale of turbidity, corresponding to approximately 3 × 10^8^ CFU/mL. Evaluation was also made against a mixed culture obtained by plating a dilution of 1/100 of collected saliva. The study was performed under aseptic conditions, avoiding any precipitation of contaminants.

Specimens (*n* = 6) with 6 mm diameter and 3 mm thickness were obtained. The culture medium of choice was Mueller-Hinton, prepared according to the manufacturer's instructions. Thus four Petri dishes of 10 cm in diameter were obtained, a plate made for each microorganism pure culture and two Petri dishes for the mixed culture. Thus, data were collected in duplicate, increasing the fidelity of the calculation. The pit in the culture medium which housed the cements was done with punch. Prior to drilling, however, the bacteria were plated with sterile swabs. After insertion and proper identification of the bodies, plates were stored in an oven for a period of 48 hours at a temperature of 35°C. After this period, the inhibition around the test samples was measured with a caliper to an accuracy of 0.05 mm. Data were tabulated and submitted to analysis of variance (ANOVA) criterion, at a significance level of 5%.

### 2.2. Sealing Ability Evaluation

The sealing ability of the temporary restorative material in different groups was analyzed by a microleakage test. Thirty bovine teeth were selected, cleaned, and distributed into 3 groups (*n* = 10). A cavity of 2.5 mm depth and 5 mm in diameter was made in the middle third of the buccal surface of each tooth, using a cylindrical diamond bur (#3131) (KG Sorensen, Cotia, São Paulo, Brazil). The cavities were restored with a single increment of the temporary restorative material of each group and immersed in distilled water for two hours. The specimens were then stored in 100% relative humidity for 48 hours.

Then, the specimens were thermocycled (500 cycles, between 5 and 55°C), dried, and sealed with two layers of nail varnish, leaving a space of about 2 mm around the restoration. Next, the specimens were bathed in paraffin at 60°C until complete waterproofing. The specimens were immersed in a solution of methylene blue 0.5% for 24 hours and then washed to remove the excess of dye.

For microleakage analysis, the specimens were cut in two halves in a sagittal direction (*n* = 20). The dye infiltration was visually evaluated and a score was attributed to each specimen, as follows: 0: absence of infiltration; 1: infiltration on less than half of dentin thickness; 2: infiltration on half of dentin thickness; 3: infiltration greater than half of dentin thickness. The data were submitted to analysis of variance (ANOVA), at a significance level of 5%.

### 2.3. Physical and Mechanical Properties Evaluation

The setting time, solubility, and compressive strength were evaluated for all groups, according to standard #30 ADA (American Dental Association). All specimens were handled at a temperature of 23 ± 1°C and relative humidity of 50 ± 2%.

### 2.4. Determination of Setting Time

Two specimens from each group were prepared using a rectangular Teflon mold of 2 mm thickness × 35 mm length × 20 mm width with a central hole of 10 mm diameter placed on a glass plate and filled with temporary restorative material. Then, a polyester strip and a glass plate were placed on the specimen surface and the assembly was pressed (hand pressure) to remove the excess of the material. One hundred and twenty seconds after preparation of the sample, the specimens were placed in an oven at 37°C and 100% relative humidity. Ninety seconds later, the samples were submerged in a container of deionized water and an indenter needle (Gilmore needle with 400 g and flat tip of 1 mm) was vertically placed on the surface of the specimen, remaining for 5 seconds. The same procedure was repeated, making indentations in the surface at intervals of 15 seconds until the time of setting has been reached. The setting time was recorded as the period of time which elapses from the start of mixing to the time when the needle fails to penetrate completely the 2 mm depth of cement.

### 2.5. Water Sorption and Solubility

Disk-shaped specimens (*n* = 5), 6 mm in diameter (*d*) and 1 mm in height (*h*), were prepared for each group. All specimens were stored in a desiccator at 37°C with silica gel and were weighed daily to verify mass stabilization (dry mass *m*_1_), which was represented by mass variations lower than 0.1 mg in any 24 h interval. After that the specimens were stored in distilled water at 37°C for 7 days to obtain the mass after saturation in water (*m*_2_).

The specimens were then placed in a desiccator again, at 37°C, and reweighed to obtain a constant dry mass (*m*_3_). Weighing was performed using an analytical balance with 0.1 mg accuracy (Mettler-Toledo, Barueri, São Paulo, Brazil). The volume (*V*) of each specimen was calculated based on the following equation: *V* = *πR* ^2^ *h* (*R* is the specimen radius). The water sorption measurements (SA) and solubility (SL) in mg mm^−3^ were calculated as follows: SA = *m*_2_ − *m*_3_/*V*, SL = *m*_1_ − *m*_3_/*V*. Data were submitted to analysis of variance (*p* < 0.05).

### 2.6. Compressive Strength Test

Five cylindrical specimens (4 mm × 6 mm) were prepared for each group by using a dimethylpolysiloxane (Speedex, Coltene, Altstätten, Canton of St. Gallen, Switzerland) mold. The molds were placed on a glass plate and filled with the temporary restorative material with a slight excess. A polyester strip and a glass coverslip were placed on the surface of the material and pressed (hand pressure) for removing excess material. The assembly was transferred to the oven and maintained at 37°C and 100% humidity for 1 hour. After this period, the specimens were flattened using #600 silicon carbide paper, removed from the mold, and stored at 37°C in deionized water for 24 hours. Then, the specimens were placed in deionized water at 23°C for at least 15 minutes before testing.

Before the mechanical test, the diameter and thickness of each specimen were measured with a digital micrometer. The specimens were placed on a universal testing machine (Emic DL 2000, São José dos Pinhais, Paraná, Brazil) by placing the flat portion of the specimen into contact with the base of the testing machine so that the compressive force was applied to the long axis of the specimen. The specimens were loaded at a compression speed of 1 mm/min. The maximum force applied when the specimen fracture was recorded and compressive strength was calculated in N/mm^2^ (MPa) according to the equation *C* = 4*F*/*π*(pi)*d* ^2^, where *F* is the fracture load and *d* is the diameter. Data were subjected to statistical analysis (analysis of variance ANOVA at a significance level of 5%).

## 3. Results

### 3.1. Antibacterial Activity Evaluation

Means and standard deviations of inhibition zones of different groups are shown in Table 1. According to analysis of variance, there were no statistical significant differences in measures of inhibition zones among groups regardless of the type of culture used.

### 3.2. Sealing Ability Evaluation

No leakage was observed in all groups. There is only pigmentation on the temporary restorative material.

### 3.3. Determination of Setting Time

Table 2 shows the average setting time obtained for the different groups. It was observed that GII and GIII groups showed lower setting times, while the GI group showed the highest, with a value of 18 minutes 37 seconds. Although there is distinction of values, this was not statistically significant, indicating that all groups were similar.

### 3.4. Water Sorption (SA) and Solubility (SL)

Since there was no difference in the masses *m*_1_, *m*_2_, and *m*_3_ for GI (control) and GIII (chloramine T), it was only possible to calculate the SA and SL for GII triclosan (SA = 1061.03 *μ*g/mm^3^) and solubility (SL = 1061.03 g/mm^3^).

### 3.5. Compressive Strength Test

Table 3 shows the mean and standard deviation values of compressive strength for the groups. According to analysis of variance there was no statistically significant difference among groups (*p* = 0.5220).

## 4. Discussion

In dental procedures, specifically in endodontic treatments, maintenance of aseptic chain becomes paramount. Such maintenance is achieved by means of an effective isolation, adequate biomechanical preparation, use of irrigation solutions, and intracanal medications. Despite this, an efficient coronary sealing is also essential to avoid any contamination of the canals. Besides, some bacteria may be residual after the endodontic treatment, collaborating for the reacutization of periapical diseases. Therefore, it would be relevant to use temporary restorative materials with properties that prevent marginal leakage and present a satisfactory antibacterial capacity.

In this study, there was no significant difference among groups regarding their antibacterial activity, against pure cultures of* E. coli*,* S. aureus,* and saliva mixed culture, although all of them showed inhibition halos with relevant measures. This fact disagrees with the results obtained by Kooper et al. [17], wherein, using the same methodology, they observed that all temporary restorative materials were ineffective regarding their antibacterial capacity.

The results of this study could be related to the inherent limitations of the performed test that could hide the expected results of the experiment groups (G2 and G3). It is understood that the inclusion of solid material (specimens) in the culture medium can hinder the diffusion of antibacterial agent included in the material's composition causing no distinction in inhibition of the microorganisms compared to the negative control group (GI). Therefore, further studies are necessary, with different methodologies, to verify the correlation of the results with respect to antibacterial capacity.

The GI group (negative control) showed a mean of 27.33 mm inhibition halo. In a previous study [16] using the same methodology the same material showed a 13 mm inhibition halo. Considering that the test conditions of this study were similar to those in [13], it is assumed that this change on measurements should result from the composition of the material itself.

Regarding the sealing ability of the groups, none showed marginal leakage (dye penetration), which denotes an efficient sealing ability of this material. However, it was observed that GII and GIII groups showed a deep pigmentation of the material, whereas in GI just a small surface layer of the material was stained. Thus, it appears that the inclusion of antibacterial agents favors a higher porosity in the material without interfering with the ability of marginal sealing. Further studies are necessary to evaluate the materials behavior in long term, in an attempt to determine whether the material's staining will not become a source of dentin infiltration.

For the maintenance of material's integrity, an adequate mechanical resistance should be present. This way, the material can withstand the loads generated during masticatory movements. In this study, the highest compressive strength was found for GII group. However, there was no statistical significant difference among groups.

The other physical-mechanical properties tested (setting time and water sorption and solubility) showed no significant changes among groups, concluding that the addition of antibacterial agents to the material's composition did not cause significant interference.

Based on the results of this study, it can be concluded that the addition of antibacterial agents on a temporary restorative material did not optimize the antibacterial ability of the material and also did not change its physical-mechanical properties.

## Figures and Tables

**Table 1:** Mean (standard deviation) of inhibition zone (mm) for specimens (*n* = 2) of the different groups.

Group	*E*. *coli*	*S*. *aureus*	Mixed culture
GI control	24 (0)	26.5 (±3.53)	31.5 (±2.12)
GII triclosan	24.5 (±0.70)	27.5 (±0.70)	32 (±5.65)
GIII chloramine T	25 (±1.41)	28 (±1.41)	33 (0)
*p* value^*∗*^	**p** = 0.6036	**p** = 0.8050	**p** = 0.9112

^*∗*^ *p* values > 0.05 indicate no statistical significant difference (ANOVA).

**Table 2:** Mean of the setting time for different groups (*n* = 2).

Group	Setting time (mean)
GI control	18′37′′
GII triclosan	16′05′′
GIII chloramine T	17′21′′

**Table 3:** Mean and standard deviation (SD) of compressive strength values for the different groups (*n* = 7).

Groups	Mean (MPa)	SD	ANOVA^*∗*^
GI control	25.41	3.83	A
GII triclosan	28.68	7.03	A
GIII chloramine T	24.37	9.70	A

^*∗*^Means followed by the same letter in the column indicate no statistically significant difference (ANOVA, *p* < 0.05).
